# MicroRNA–Ferroptosis–Spinal Cord Injury: A Complex Interplay in Neurodegeneration and Repair

**DOI:** 10.3390/ijms27177711

**Published:** 2026-08-28

**Authors:** Raju Poongodi, Tao-Hsiang Yang, Kuender D. Yang, Hsin-Chieh Lin, Jen-Kun Cheng

**Affiliations:** 1 Department of Medical Research, MacKay Memorial Hospital, Taipei 104217, Taiwan; poongodiraju@gmail.com (R.P.); konnankonnan@yahoo.com.tw (T.-H.Y.); yangkd.yeh@hotmail.com (K.D.Y.); 2 MacKay Children’s Hospital, Taipei 104217, Taiwan; 3 Institute of Clinical Medicine, National Yang Ming Chiao Tung University, Taipei 112304, Taiwan; 4 Department of Materials Science and Engineering, National Yang Ming Chiao Tung University, Hsinchu 300093, Taiwan; hclin45@nycu.edu.tw; 5 Center for Intelligent Drug Systems and Smart Bio-Devices (IDS2B), National Yang Ming Chiao Tung University, Hsinchu 300093, Taiwan; 6 Department of Anesthesiology, MacKay Memorial Hospital, Taipei 104217, Taiwan; 7 Department of Medicine, MacKay Medical University, New Taipei City 252005, Taiwan

**Keywords:** microRNAs, ferroptosis, spinal cord injury, neurodegeneration, neuroprotection, lipid peroxidation, iron accumulation, ferritinophagy, oxidative stress, extracellular vesicles

## Abstract

Spinal cord injury (SCI) is a devastating neurological condition characterized by irreversible primary damage followed by a complex secondary injury cascade involving oxidative stress, neuroinflammation, iron dysregulation, and regulated cell death. Among these mechanisms, ferroptosis, a distinct, iron-dependent form of regulated cell death driven by lipid peroxidation and redox imbalance, is increasingly recognized as a critical mediator of neurodegeneration after SCI. The miRNAs play essential roles in neural injury responses by modulating inflammation, oxidative stress, and cell-death pathways. Growing evidence indicates that miRNAs closely regulate ferroptosis-related signaling networks following SCI, influencing key molecular targets including iron metabolism, antioxidant defense systems, and lipid peroxidation pathways. Conversely, ferroptotic stress may alter miRNA expression profiles, suggesting a bidirectional regulatory relationship. In addition, ferritinophagy, a selective autophagy pathway degrading ferritin via nuclear receptor coactivator 4 (NCOA4), has emerged as an important yet underexplored regulator of intracellular iron homeostasis and ferroptosis susceptibility in SCI. This review systematically summarizes current evidence on the molecular mechanisms linking miRNAs and ferroptosis in SCI, highlights how miRNA-mediated regulation of ferroptosis contributes to neuronal death, glial responses, and impaired regeneration, and discusses emerging therapeutic strategies targeting this axis to promote neuroprotection and functional recovery. By integrating recent experimental findings, we aim to provide mechanistic insight and identify translational opportunities for miRNA- and ferroptosis-based interventions in SCI.

## 1. Introduction

**Figure 1:**
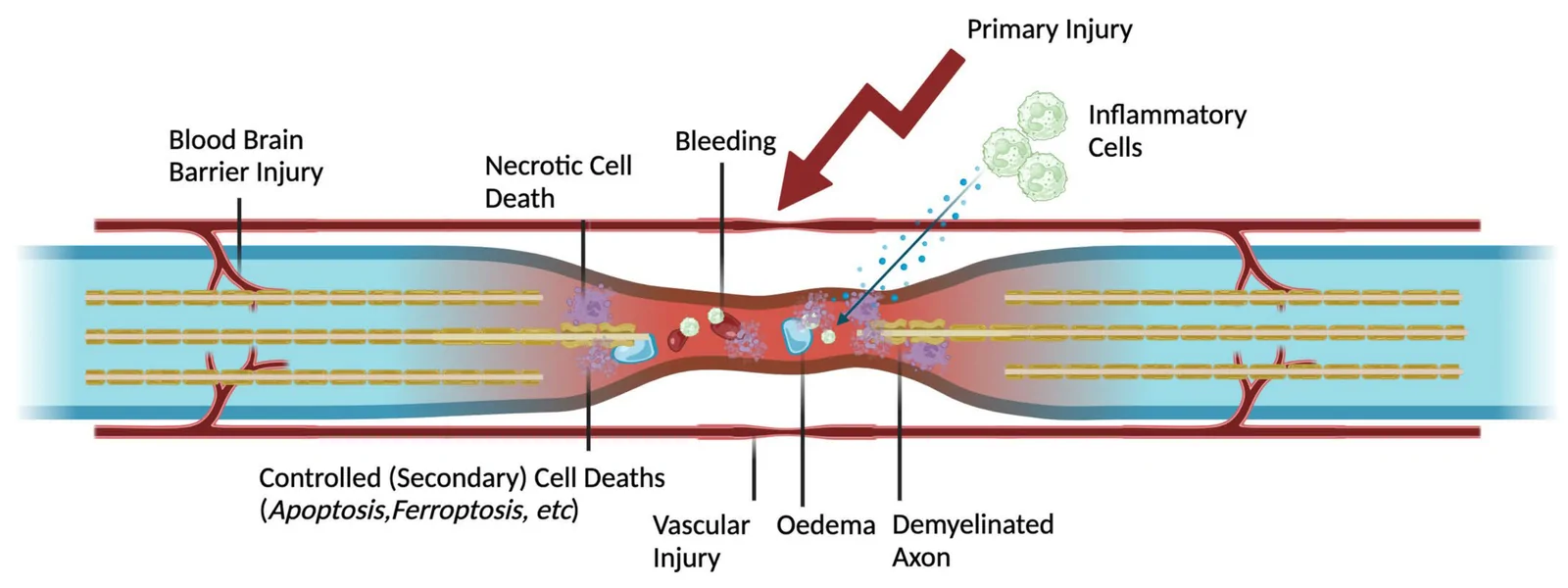
Spinal cord injury involves various physiological and pathological changes that occur during the acute phase of the injury. Reprinted with permission from [6].

Spinal cord injury (SCI) is a devastating insult to the central nervous system (CNS) that results in profound and often permanent loss of sensory, motor, and autonomic function below the level of injury [1]. This injury imposes severe functional deficits and immense financial and psychological burdens on affected individuals and society [2]. Figure 1 shows the pathophysiology of SCI is characterized by a biphasic process: the primary injury involving direct mechanical damage, followed by a secondary injury cascade of biochemical and cellular events that exacerbate initial damage over time [3].

The hallmarks of SCI include neuronal loss, axonal degeneration, reactive gliosis, and a pronounced neuroinflammatory response [4]. A significant challenge in treating SCI lies in the CNS’s inherently limited regenerative capacity, making spontaneous neuronal recovery rare after injury [5]. This lack of intrinsic repair mechanisms underscores the need to explore novel therapeutic strategies that protect the remaining neural tissue or actively promote the survival and regeneration of damaged neurons.

MicroRNAs (miRNAs) are a class of small, non-coding RNA molecules, typically 20–24 nucleotides in length, that play a critical role in the post-transcriptional regulation of gene expression [7]. They exert their regulatory effects by binding to the 3′-untranslated region (3′-UTR) of target messenger RNAs (mRNAs), leading to either the inhibition of protein translation or the degradation of the mRNA transcript [7]. These small RNA molecules are involved in a wide array of biological processes that are highly relevant to SCI pathology, including inflammation, oxidative stress, apoptosis, and neurodevelopment [8]. Given their broad regulatory roles in processes critical to SCI, miRNAs are recognized as key players in the disease and potential therapeutic targets.

Ferroptosis represents a distinct form of regulated cell death, characterized as non-apoptotic and dependent on iron, with a primary feature being the peroxidation of lipids [9]. Key factors in this cell death mechanism include reliance on ferric ions, accumulation of reactive oxygen species (ROS), iron overload, and lipid peroxidation [2,10]. Morphologically, cells undergoing ferroptosis exhibit characteristic changes such as mitochondrial shrinkage, increased membrane density, and a reduction in or absence of mitochondrial cristae [11]. The process of ferroptosis is tightly regulated by key molecules, including glutathione peroxidase 4 (GPX4) and ferroptosis suppressor protein 1 (FSP1), which function to inhibit this form of cell death [12]. Fsp1, previously referred to as apoptosis-inducing factor 2 (Aifm2), has been highlighted in studies [13,14] as a key component that works in conjunction with GPX4. Given its unique mechanisms and morphological features, which are distinct from those of other cell-death pathways, ferroptosis represents a specific and potentially crucial target for therapeutic intervention in SCI.

Emerging research suggests a potential interplay between miRNAs and ferroptosis in the context of SCI, indicating that these two biological processes may be interconnected and contribute to both neurodegeneration and the potential for repair following SCI. This review aims to explore this complex interaction, synthesizing current findings from various research articles to provide a thorough understanding of the existing knowledge in this field. The scope of this report includes an in-depth analysis of the molecular mechanisms involved in this interplay, the evidence supporting their interaction in the context of SCI, and the therapeutic implications of targeting this axis for the treatment of SCI.

## 2. Extracellular Vesicles, MicroRNA, and Ferroptosis

**Figure 2:**
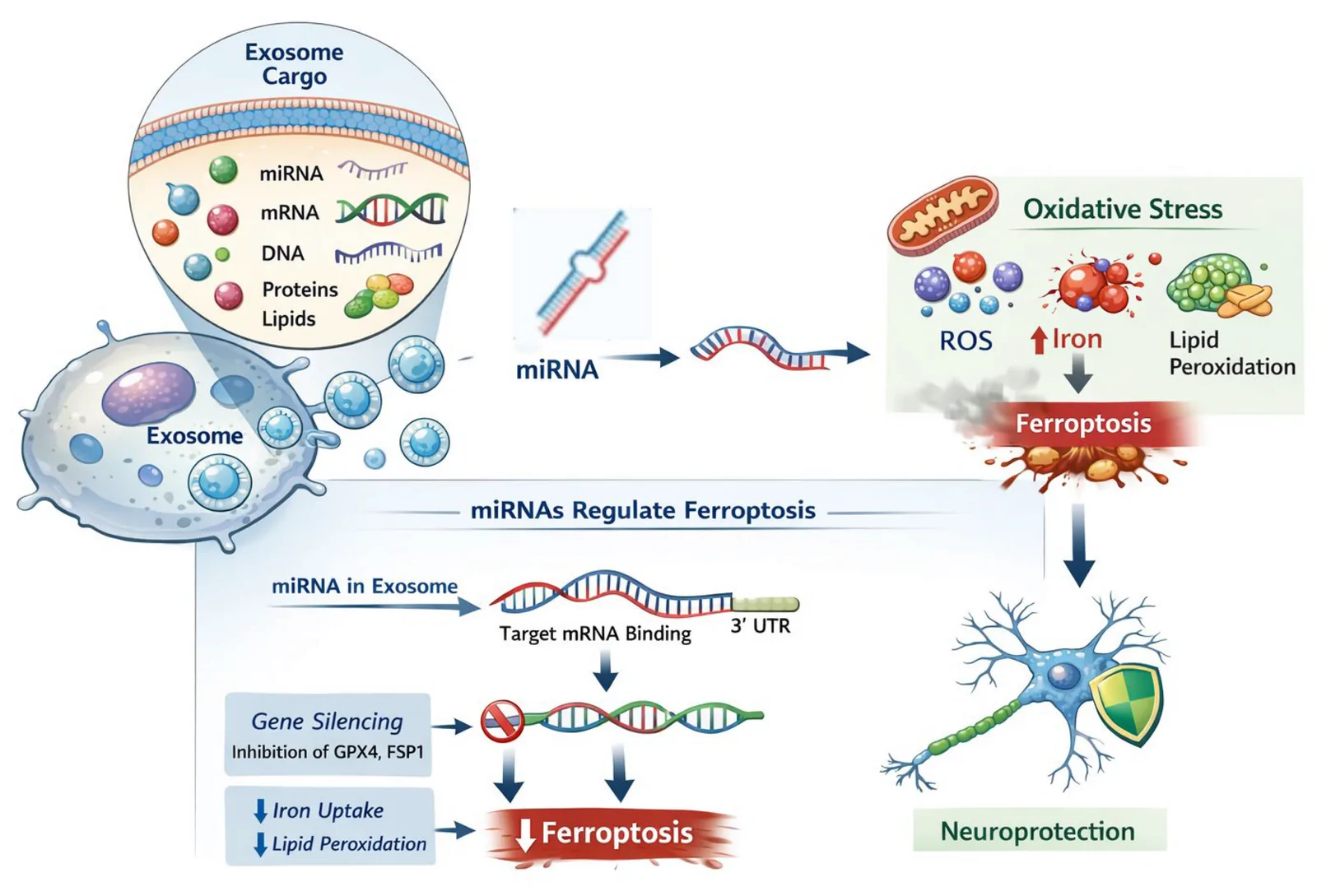
Role of extracellular vesicle (EV)-derived miRNAs in regulating ferroptosis. EVs are nanosized extracellular vesicles that mediate intercellular communication by transferring bioactive cargo, including miRNAs, mRNAs, proteins, lipids, and DNA. Following neural injury, EV-derived miRNAs are internalized by recipient cells and regulate gene expression through binding to the 3′ untranslated region (3′UTR) of target mRNAs, resulting in translational repression or mRNA degradation. These miRNAs influence key ferroptosis-related processes, including iron uptake, lipid peroxidation, and antioxidant capacity. By modulating ferroptosis signaling pathways, EV miRNAs contribute to the regulation of neuronal survival and may represent a promising therapeutic strategy for mitigating ferroptosis-mediated damage in spinal cord injury.

Extracellular vesicles (EVs) have emerged as critical, highly coordinated vehicles for cell-to-cell communication within the CNS [15]. These membrane-bound structures encapsulate diverse bioactive molecular payloads, including genomic DNA, messenger RNAs (mRNAs), microRNAs (miRNAs), structural lipids, and signaling proteins [16]. By serving as protective envelopes, EVs transport these regulatory molecules safely through the extracellular space to recipient cells. miRNAs are critical regulators of fundamental cellular processes, including proliferation, differentiation, signaling, cell growth, and cell death (Figure 2).

Among the distinct subpopulations of EVs, exosomes—nanosized vesicles ranging from approximately 30 to 150 nm in diameter—play highly specialized roles in maintaining neural tissue homeostasis and coordinating multicellular responses to pathological stress [17]. Secreted dynamically by neurons, astrocytes, microglia, and oligodendrocytes, exosomes cross biological barriers to modulate synaptic plasticity, direct immunomodulatory pathways, stimulate local angiogenesis, and promote neurogenesis [18]. Under physiological conditions, their cargo carefully regulates critical signaling cascades required for neural development. However, following trauma, these exosomal communication networks shift, often delivering altered instructions that modulate inflammation, autophagy, oxidative stress, and cell-death pathways within the surrounding injury microenvironment [19].

MicroRNAs transferred via EVs act as post-transcriptional master regulators of recipient cell transcriptomes. Under standard physiological constraints, stable baseline miRNA expression is essential for orchestrating vital cellular processes, including proliferation, structural differentiation, and cell survival [20]. Conversely, abnormal variations in miRNA synthesis, sorting, or activity are fundamentally linked to the development and progression of diverse neurodegenerative disorders and systemic diseases [20].

Mechanistically, once an EV or exosome fuses with internalized by a recipient cell, its regulatory miRNA cargo is released directly into the cytoplasm [21]. These mature single-stranded miRNAs track and bind sequence-specifically to the 3′-untranslated regions (3′-UTRs) of complementary target messenger RNAs (mRNAs) [22]. Depending on the degree of sequence complementarity, this binding recruits the RNA-induced silencing complex (RISC), resulting in either translational repression or direct mRNA degradation [21]. Because a single miRNA can target multiple genes across interconnected metabolic pathways, exosomal miRNA delivery offers a powerful mechanism to simultaneously silence detrimental pro-death machinery or upregulate protective survival networks within injured tissues (Figure 2).

Importantly, research indicates that the abnormal expression of miRNAs is associated with the development and progression of many diseases [23,24]. Therefore, modulating the production and activity of these miRNAs holds potential for therapeutic intervention in a wide range of diseases. The cellular response to oxidative stress is a crucial factor in determining cell fate. ROS, oxygen-derived free radicals generated during metabolism, can induce lipid peroxidation, ultimately leading to ferroptosis [25]. This occurs when agents such as erastin and RAS-selective lethal compound 3 (RSL-3) further drive lipid peroxidation beyond what the cellular antioxidant system can counteract, activating the genes and pathways that promote ferroptotic cell death [26,27]. EVs also encapsulate miRNAs that regulate cellular functions beyond ferroptosis; recent work has linked EV-derived miRNAs to pyroptosis, another form of regulated cell death [28]. In a study by Xia et al., EVs from adipose-derived stem cells (ADSCs) overexpressing miR-19b-3p were shown to reduce ferroptosis and neuronal damage following intracerebral hemorrhage. In essence, EVs loaded with regulatory miRNAs play a critical role in mediating intercellular communication. Given that miRNAs regulate key cellular functions and their dysregulation contributes to disease pathogenesis, they represent promising therapeutic targets.

## 3. Role of MicroRNAs in Pathway-Related Spinal Cord Injury-Induced Ferroptosis

Numerous miRNAs show altered expression levels in animal models following SCI [29]. These changes are not static; certain miRNAs are upregulated, downregulated, or show bidirectional changes at different time points after injury [30]. This dynamic regulation suggests that miRNAs are involved in various phases of the injury and repair processes, highlighting the importance of considering the temporal context when exploring them as therapeutic targets.

Several specific miRNAs have been identified as playing critical roles in the pathological events that unfold following SCI. In the context of inflammation and immune responses, miRNA-543-5p and miRNA-940 have been shown to regulate inflammation [31,32]. Notably, miRNA-155 acts as a proinflammatory miRNA, contributing to the M1 polarization of microglia, a phenotype associated with the release of proinflammatory cytokines [33]. Conversely, the downregulation of miRNA-24-3p after SCI has been observed, and its upregulation or the knockdown of MAPK-activated protein kinase 2 (MK2) has been shown to reduce microglial cell activation and the expression of inflammatory cytokines such as TNF-α and IL-1β [3]. Furthermore, miRNA-146a is upregulated following spinal cord contusion [3], and miR-92a is induced in inflamed spinal cord neurons, where it targets cytoplasmic polyadenylation element-binding protein 3 (Cpeb3) [34].

Additionally, miRNA-125a and miRNA-216a-5p regulate the polarization of microglia towards the M1 or M2 phenotype, thereby inhibiting the overall inflammatory response after SCI [11]. miR-129-5p has also been shown to alleviate SCI by suppressing inflammation through the High-Mobility Group Protein B1 (HMGB1)/Toll-like receptor 4 (TLR4)/Nuclear factor-κB (NF-κB) pathway [35]. These findings demonstrate that specific miRNAs act as key regulators of the inflammatory response following SCI, presenting potential targets for modulating neuroinflammation and its detrimental effects (Table 1).

Oxidative stress represents another critical, destructive component of the secondary injury cascade in SCI, and altered miRNA profiles have been consistently linked to this process [3]. Specifically, miRNA-99a and miRNA-672-3p have been shown to regulate oxidative stress induced after SCI in rat models [39]. This evidence suggests that specific miRNAs actively participate in the complex cellular redox imbalance that occurs post-SCI, directly influencing the accumulation of damaging ROS.

Furthermore, miRNAs are intricately involved in the regulation of various forms of cell death after SCI, including apoptosis and ferroptosis [3]. For instance, miRNA-9-5p and miRNA-488 are involved in the regulation of neuronal apoptosis [40,41], and miR-21 has been shown to regulate both proliferation and apoptosis in neural progenitor cells [42]. Notably, miRNA-124 has demonstrated the ability to suppress apoptosis of neuronal cells, leading to improved functional recovery [43]. In contrast, miRNA-372 has been implicated in inhibiting SCI recovery, potentially through its involvement in inflammatory signaling [44]. These findings highlight that miRNAs are crucial regulators of cell-death pathways following SCI, making them attractive targets for neuroprotective strategies.

Beyond inflammation and cell death, miRNAs also play a role in the structural remodeling of the spinal cord after injury, influencing axonal regeneration and the formation of the glial scar [5]. For example, miR-219 has the potential to promote the differentiation of oligodendrocytes, which are essential for the process of remyelination [45]. This indicates that miRNAs can affect the structural changes occurring in the SCI, impacting the regeneration of nerve fibers and the glial scar, which can either facilitate or impede recovery depending on its characteristics.

Given their specific actions and involvement in these key pathological processes, miRNAs represent promising potential therapeutics for the treatment of SCI. The concept of miRNA replacement therapy aims to deliver miRNAs to diseased cells to promote repair processes [5]. However, the clinical translation of miRNA-based therapies faces significant challenges, including the need to optimize dosages, delivery mechanisms to the target site in the spinal cord, and to ensure long-term safety profiles.

### MicroRNA Landscape in SCI: Recent Advances

The miRNA landscape in SCI is highly dynamic, with expression profiles shifting throughout acute and chronic stages [20]. Beyond well-known regulators, recent research has identified new candidates:

1. miR-5627-5p: Delivered via MSC-derived EVs, this miRNA inhibits neuronal ferroptosis through the miR-5627-5p/FSP1 axis, enhancing cellular resistance to oxidative death [46].
2. miR-9-3p: Recently identified in cerebrospinal fluid (CSF) EVs as a biomarker and therapeutic target that adapts neuronal energy metabolism and reduces stress responses post-SCI [47].
3. miR-133b: A gold standard for promoting axonal regeneration by targeting the RhoA pathway. Recent trials using miRNA-loaded EVs at 200 μg dosage showed optimal functional recovery in spinal cord contusion models [48].

## 4. Ferroptosis as a Critical Factor in Spinal Cord Injury

Iron dependency is a central characteristic, where iron overload can enhance lipid peroxidation and ROS production, potentially triggering ferroptosis [12]. Following SCI, iron derived from red blood cells and damaged cells accumulates at the injury site [49]. This iron, particularly within the labile iron pool (LIP), can participate in the Fenton reaction (the iron-catalyzed reduction of hydrogen peroxide, Fe^2+^ + H_2_O_2_ → Fe^3+^ + •OH + OH^−^, that generates the hydroxyl radical), generating highly reactive hydroxyl radicals that initiate lipid peroxidation [50]. The homeostasis of iron within cells is tightly controlled by proteins such as transferrin receptor (TfR) and ferritin, and this regulation is influenced by iron regulatory proteins (IRPs) [50].

SCI can cause brain iron buildup, activating microglia via NF-κB, which releases inflammatory substances that damage neurons and contribute to central pain. Reducing iron or inhibiting NF-κB/microglia might ease SCI-induced central pain [51]. Feng et al. studied the activated microglia in the motor cortex cause iron overload in motor neurons, triggering ferroptosis and impeding motor recovery following SCI [52]. Targeting microglial activation and iron overload presents potential therapeutic strategies for SCI [52].

A primary driver of ferroptosis is the excessive accumulation of intracellular lipid peroxides. Polyunsaturated fatty acids (PUFAs) present in cell membranes are particularly vulnerable to this process [12]. Enzymes, such as acyl-CoA synthetase long-chain family member 4 (ACSL4) and lipoxygenases (LOXs), play a role in promoting this lipid peroxidation [53]. The regulation of ferroptosis involves key proteins. GPX4, a critical antioxidant enzyme, protects cells from ferroptosis by reducing lipid peroxides, utilizing glutathione (GSH) as a necessary cofactor [54]. FSP1 provides an alternative, glutathione-independent pathway to inhibit ferroptosis by reducing ubiquinone (CoQ10) [55]. The intricate balance between iron metabolism, lipid peroxidation, and these antioxidant defense mechanisms ultimately determines the susceptibility of cells to ferroptosis, offering multiple potential targets for therapeutic intervention in SCI.

A cell type-specific perspective is important here, since susceptibility to ferroptosis is not uniform across the spinal cord. Oligodendrocytes, the myelin-producing glia, appear particularly vulnerable: they contain lower glutathione levels than astrocytes, leaving the GSH/GPX4 system less capable of keeping pace with lipid peroxidation, and oligodendrocyte precursor cells carry a substantially higher total iron content than astrocytes, both of which favor ferroptosis induction [56]. Myelin itself is markedly lipid-rich, comprising roughly 80% lipid by dry weight, and myelin debris generated after SCI is a recognized source of peroxidizable lipid at the injury site. Infiltrating macrophages that phagocytose myelin debris accumulate lipid to the point of becoming foam cells, and unresolved myelin-derived lipid is linked to oxidative damage and chronic inflammation in the injured cord [40]. Whether this myelin-derived lipid pool is oxidized specifically through the iron- and GPX4-regulated ferroptosis pathway, as opposed to other oxidative or inflammatory routes, has not been directly tested and represents an open question for this field; if confirmed, it would position myelin breakdown as both a consequence of oligodendrocyte ferroptosis and a potential feed-forward substrate for further lipid peroxidation in the surrounding tissue.

A substantial amount of evidence supports the involvement of ferroptosis in the pathogenesis of SCI, derived from both in vitro and in vivo research [2]. The presence of iron overload, the accumulation of ROS, and lipid peroxidation, all hallmarks of ferroptosis, have been consistently observed in the context of SCI [12]. Ferroptosis is considered a major contributor to the secondary injury that occurs after the initial spinal cord trauma [57]. Studies have indicated that ferroptosis occurs in distinct phases following SCI, with an acute phase occurring within the first two days post-injury and a subacute phase extending from three to fourteen days after the injury [58]. Table 2 summarizes the temporal profile of ferroptosis after SCI, including key molecular events and biomarkers observed at different post-injury stages. The temporal profile suggests that ferroptosis plays a critical role in the early stages of secondary damage.

The process of ferroptosis significantly contributes to cell death following SCI, impacting both neurons and oligodendrocytes [61]. The ferroptosis-induced mitochondrial atrophy and functional decline further disrupt the intricate electrical and chemical signaling processes within neurons [62]. Given the significant role of ferroptosis in the detrimental processes after SCI, therapeutic strategies targeting this specific form of cell death have been explored. Inhibiting ferroptosis after SCI has been shown to aid in functional recovery in preclinical studies. Several ferroptosis inhibitors, including ferrostatin, deferoxamine, and SRS16-86, have demonstrated neuroprotective effects and improved outcomes in animal models of SCI [12]. Furthermore, natural compounds such as proanthocyanidins, epigallocatechin gallate (EGCG), carnosic acid, and trehalose have also shown promise in inhibiting ferroptosis and promoting recovery following SCI [63]. Specifically, EGCG inhibits ferroptosis by enhancing GPX4 via the ERK1/2 pathway and, administered intraperitoneally at 50 mg/kg, reduces the inflammatory markers TNF-α, IL-1β, and iNOS in rat SCI models; a proanthocyanidin-rich fraction, given intraperitoneally, has been shown to preserve GSH/GPX4 levels, limit iron accumulation, and reduce lipid peroxidation in a mouse SCI model; and trehalose activates the Nrf2/HO-1 pathway to inhibit ferroptosis and ferroptosis-related inflammation in a mouse SCI model. Carnosic acid, by contrast, has so far only been validated against ferroptosis in erastin-treated PC12 cells and has not yet been tested directly in an SCI animal model. These findings highlight the potential of targeting ferroptosis as a therapeutic approach for mitigating the damage caused by SCI and enhancing functional outcomes.

## 5. Delivery Methods for Ferroptosis Inhibitors in Spinal Cord Injury Treatment

### 5.1. Localized Injection at the Traumatized Spinal Cord

In preclinical studies, researchers have directly injected ferroptosis inhibitors, such as ferrostatin-1, into the injured spinal cord of rats. Ge et al. microinjected ferrostatin-1 (10 μL per site) into the dorsal spinal cord at 2 mm rostral and 2 mm caudal to the contusion epicenter, with doses delivered on post-injury days 1 and 2. This regimen reduced iron and ROS accumulation, downregulated the ferroptosis-associated transcripts IREB2 and PTGS2 in oligodendrocytes, and suppressed reactive astrocyte and microglial activation; histological assessment showed decreased white matter injury, and functional recovery, measured with the BBB locomotor rating scale, was modestly improved relative to vehicle-treated controls [64]. Similarly, a single local injection of sodium selenite (3 μL, 2.5 μM) into the injury site 10 min after T10 contusion lowered iron, MDA, and 4-HNE levels while upregulating SP1 and GPX4 expression, promoting neuronal and oligodendrocyte survival and yielding better BBB locomotor scores than vehicle-treated controls [65]. While precise, this method’s clinical application is challenging due to manipulation difficulties and the risk of causing further injury [66].

### 5.2. Intraperitoneal Injection

Some studies have shown that intraperitoneal injections of DFO (iron chelator deferoxamine) [67] and SRS16-86 (a third-generation ferroptosis inhibitor) [68] have both been explored as systemic treatments for SCI-associated ferroptosis. Yao et al. administered DFO intraperitoneally at 100 mg/kg 30 min before injury and daily for 7 consecutive days in a rat T10 contusion model; this regimen lowered spinal cord iron content, increased GPX4, xCT, and glutathione expression, increased neuronal survival, and produced better BBB locomotor scores than untreated SCI controls. Zhang et al. administered SRS16-86 intraperitoneally in a rat contusion model, which upregulated GPX4, glutathione, and xCT, lowered the lipid peroxidation marker 4-HNE, reduced the inflammatory markers IL-1β, TNF-α, and ICAM-1, attenuated astrogliosis, and improved BBB locomotor scores relative to controls. Together, these findings indicate that both agents inhibit ferroptosis by upregulating the Xc–GSH–GPX4 axis. However, more research is needed to determine if these drugs can effectively cross the BBB or blood spinal cord barrier (BSCB) for clinical use.

### 5.3. Intranasal Administration

Intranasal administration offers a promising route as it can bypass the BBB/BSCB and deliver drugs directly to the CNS in animal models [69]. This route uses the olfactory and trigeminal nerve pathways to bypass first-pass hepatic metabolism and reduce systemic exposure. For instance, ferrostatin-1 and liproxstatin-1 administered intranasally at 10 mg/kg immediately after injury reduced infarct volume in a mouse cerebral ischemia (MCAO) model [70]. This evidence comes from a cerebral ischemia model rather than SCI itself, so whether nose-to-brain delivery can reach the spinal cord, which lies further from the nasal cavity than the brain, remains to be directly established; efficient delivery may depend on cerebrospinal fluid bulk flow along perivascular and perineural pathways, and effective dosing for spinal targets may need to be higher or more frequent than for cerebral targets. The main limitations of this route are its comparatively low and variable bioavailability, dependence on the drug’s molecular size and lipophilicity, mucociliary clearance in the nasal passage, and the current absence of direct validation in an SCI model.

### 5.4. Intravenous Injection

Intravenous injection is a highly practical option for emergency clinical treatment due to its ease of use, ability to deliver large drug volumes, and low risk. However, its effectiveness in crossing the blood–brain barrier (BBB) for CNS diseases remains a subject of debate. For example, intravenous SRS16-86 (a third-generation ferroptosis inhibitor) did not cross the BBB in mice, as it was undetectable in cerebrospinal fluid and brain tissue fluid after the injection [68]. This illustrates that many small-molecule ferroptosis inhibitors are poor candidates for intravenous delivery to the CNS without chemical modification or a barrier-penetrant carrier, such as a nanoparticle or peptide conjugate, to improve BBB/BSCB permeability. Taken together, these four delivery strategies illustrate a trade-off between anatomical precision and clinical practicality. Localized injection directly at the injury site achieves the highest local drug concentration and has produced the most consistent molecular and functional benefits among the agents discussed above, but its invasiveness and risk of secondary damage restrict its use largely to research settings. Intraperitoneal injection is technically simple and can deliver systemic drug levels sufficient to inhibit ferroptosis, as shown for DFO and SRS16-86, but its utility is limited by uncertain and compound-dependent BBB/BSCB penetration. Intranasal administration offers a non-invasive route that bypasses the BBB, but its efficacy has so far only been demonstrated for cerebral rather than spinal targets, and translating this approach to SCI will require confirming that nose-to-spinal cord delivery is feasible. Intravenous injection is the most practical and scalable route for acute emergency treatment, but as shown for SRS16-86, its inability to cross the BSCB substantially limits its usefulness for centrally acting ferroptosis inhibitors unless paired with barrier-penetrant formulations. Overall, no single route currently satisfies both efficacy and clinical practicality: local and intraperitoneal administration currently offer the strongest preclinical evidence for ferroptosis inhibition in SCI, while intranasal and intravenous strategies remain promising but underexplored avenues that will require SCI-specific validation and barrier-penetration engineering before clinical translation.

## 6. Mitigating Ferroptosis: The Role of Extracellular Vesicles in Spinal Cord Injury Recovery

EVs present a promising alternative to MSCs in therapeutic applications, offering advantages in safety, controllability, and logistical handling [71]. In SCI, EVs exert therapeutic effects through diverse mechanisms, including neuroprotection [72], stimulation of nerve tissue regeneration [73,74], attenuation of scar formation [75,76], and regulation of oxidative stress and angiogenesis [77]. Notably, MSC-derived EVs have been shown to reduce oxidative stress and restore the BBB [78], as well as inhibit neuronal cell-death pathways, apoptosis [79], and ferroptosis [80]. Furthermore, EVs can function as efficient drug carriers due to their inherent properties of small size, high tissue penetration, and avoidance of immune clearance [81]. These therapeutic actions are mediated by suppressing detrimental pathological responses and activating regenerative signaling pathways in neural and vascular tissues [78]. The future direction of SCI therapy is leaning towards combination approaches [79], where the integration of EVs with degradable bio-scaffolds has demonstrated synergistic effects, enhancing treatment efficacy and facilitating nutrient delivery to the injury site [82].

Exosomes, tiny vesicles secreted by cells, are being extensively studied as natural carriers for therapeutic molecules like miRNAs (Table 3). Their ability to cross the BBB and protect their cargo from degradation makes them ideal for SCI treatment [83]. By modulating specific miRNAs, exosomes can influence lesion size, cellular dynamics, and functional recovery [84]. Preclinical studies have identified several exosomal miRNAs that can inhibit ferroptosis in SCI models. For example, MSC-derived exosomes carrying miR-219-5p have shown the ability to reduce ferroptosis in neuronal cells and improve motor function in SCI rats by targeting pathways like UBE2Z/NRF2 [85]. Other miRNAs, such as miR-125a-3p, have been shown to alleviate SCI by regulating neutrophil extracellular trap formation, which contributes to inflammation and hinders recovery [57].

Exosomal miRNAs can exert their beneficial effects through various pathways beyond direct ferroptosis inhibition, including reducing inflammation, promoting neuroprotection, enhancing axonal regeneration, and influencing macrophage/microglia polarization [86]. Overall, EVs may carry molecules that either inhibit factors that promote ferroptosis or enhance those that prevent it, and they can deliver factors linked to iron homeostasis and lipid peroxidation. However, because EVs exert broad, pleiotropic effects on inflammation, apoptosis, and regeneration (Table 3), the functional and histological improvements reported in these studies cannot be attributed specifically to ferroptosis modulation without dependency—or rescue–experiments, such as co-administering a ferroptosis inducer or genetically restoring ferroptosis susceptibility alongside the EV treatment, that isolate the ferroptosis contribution from these other mechanisms. In the absence of such experiments, the association between EV therapy and reduced ferroptotic markers should be read as correlational rather than as evidence that ferroptosis suppression is the operative mechanism of functional recovery.

## 7. Unraveling MicroRNA–Ferroptosis Crosstalk in Spinal Cord Injury

Emerging evidence suggests a crucial link between miRNAs and ferroptosis in SCI [87]. These miRNAs act through several converging mechanisms. For instance, some miRNAs can directly target genes involved in iron uptake and storage, thereby influencing cellular iron levels and susceptibility to ferroptosis [49]. Others can modulate the expression of genes involved in GSH synthesis or GPX4 activity, impacting the cellular antioxidant capacity and resistance to lipid peroxidation [68]. Furthermore, miRNAs can indirectly influence ferroptosis by targeting other signaling pathways involved in oxidative stress and inflammation, which are closely intertwined with ferroptosis [67].

One notable example is miR-672-3p, which is upregulated after SCI. Studies have demonstrated that this miRNA promotes ferroptosis by downregulating the expression of FSP1 [3]. FSP1 normally functions to inhibit ferroptosis, and its downregulation by miR-672-3p impairs this protective mechanism, leading to increased lipid peroxidation and ferroptotic cell death in the injured spinal cord. Conversely, inhibiting miR-672-3p has been shown to suppress ferroptotic cell death and alleviate the damage that occurs after SCI [36]. This suggests that miR-672-3p plays a significant role in promoting ferroptosis in SCI by targeting FSP1, and its inhibition could represent a potential therapeutic strategy.

Another important miRNA is miR-6315, which is also significantly upregulated following spinal cord injury. Research indicates that miR-6315 negatively regulates Smoothened (Smo), a key factor in the axonal growth cone pathway, as well as components of the anti-ferroptosis pathway, including xCT, GSH, and GPX4 [37]. Silencing miR-6315 in animal models of SCI has been shown to improve functional recovery, promote neuronal regeneration and migration, and attenuate both cell apoptosis and ferroptosis [37]. This suggests that miR-6315 has a detrimental role in SCI by suppressing neuroprotective pathways and promoting ferroptosis, making its silencing a promising therapeutic approach.

In addition, let-7b-5p has been identified as part of a ferroptosis-related mRNA-miRNA-lncRNA network in SCI [3]. Similarly, miR-15b-5p has also been found within this ferroptosis regulatory network in SCI [38]. One more study revealed the exosomes derived from iPSC-NSCs can package and deliver let-7b-5p to downregulate LRIG3, thereby alleviating microglia/macrophage pyroptosis and improving motor function in mice post-SCI [88]. These findings underscore the therapeutic potential of combining iPSC-NSC-derived exosomes with let-7b-5p to enhance functional recovery and reduce inflammation after SCI [88]. These findings suggest that both let-7b-5p and miR-15b-5p may play roles in modulating ferroptosis in the context of SCI, although further research is needed to fully elucidate their specific targets and functions within this network. Shao et al. investigated alternative cell-death pathways, including ferroptosis, using MSC-derived EVs. They demonstrated that exosomal lncGm36569 acts as a competing endogenous RNA for miR-5627-5p, thereby inhibiting neuronal ferroptosis via the miR-5627-5p/FSP1 axis and reducing neuronal dysfunction [46].

Beyond these, several miRNAs implicated in SCI (such as miR-21, miR-124, miR-940, and miR-26a) may indirectly influence ferroptosis through their established roles in regulating inflammation, apoptosis, and oxidative stress [3]. The interplay between miRNAs and ferroptosis in SCI is likely a complex and multifaceted process involving numerous miRNAs with diverse targets and functions. A comprehensive understanding of the entire network of miRNAs and their interactions with the ferroptosis pathway is essential for developing effective therapeutic strategies (Table 4). Importantly, the relationship between miRNAs and ferroptosis is likely bidirectional. While miRNAs regulate ferroptosis, ferroptotic stress may also alter miRNA expression profiles in the injured spinal cord. Ferroptosis-induced cellular damage can trigger the release of damage-associated molecular patterns (DAMPs) and promote inflammation, both of which can influence miRNA expression. Investigating this reciprocal regulation may provide deeper insights into SCI pathophysiology.

Long non-coding RNAs (LncRNAs) further add complexity to this regulatory landscape. LncRNAs are key modulators of gene expression and are implicated in various human diseases [95]. Recent studies indicate that LncRNAs play critical roles in regulating ferroptosis and are dysregulated in SCI [96]. For instance, lncRNA OIP5-AS1 has been shown to suppress ferroptosis and protect neural cells via the miR-128-3p/Nrf2 axis, highlighting its therapeutic potential [97]. Supporting evidence from cancer studies demonstrates that OIP5-AS1 can inhibit ferroptosis through the miR-128-3p/SLC7A11 pathway [98]. In vivo experiments using lentiviral-mediated overexpression of OIP5-AS1 in SCI rat models further confirmed its neuroprotective and anti-ferroptotic effects [97].

Overall, the combined effects of miRNA dysregulation and ferroptosis on neurodegeneration and repair following SCI are highly complex and context-dependent. Certain miRNAs may exacerbate injury by promoting ferroptosis, whereas others may confer neuroprotection by inhibiting it. The balance of these opposing interactions ultimately determines functional outcomes after SCI. Therefore, elucidating the precise roles of miRNA–ferroptosis interactions is crucial for designing targeted therapeutic strategies aimed at promoting neuroprotection and regeneration.

A methodological caveat applies to the synthesis presented above and summarized in Table 4. As shown in the model system column, none of the five miRNA–target relationships cataloged have yet to be directly demonstrated in an SCI model. The evidence instead comes from a hemorrhage-exposed neuronal cell line, hepatoma and erythroid cells, and traumatic brain injury. These mechanisms are incorporated into the SCI-specific model proposed here because they converge on ferroptosis regulatory nodes, GPX4, ACSL4, SLC7A11/xCT, ATF4, and FSP1, that are broadly conserved across cell types and pathological contexts, making cross-context extrapolation a reasonable starting hypothesis. However, conservation of a molecular target does not guarantee that a given miRNA regulates it the same way, at the same magnitude, or with the same functional consequence in the injured spinal cord as in cancer, hemorrhage, or non-neural tissue. The unified miRNA–ferroptosis axis in SCI proposed in this review should therefore be read as a mechanistically motivated hypothesis assembled from cross-context evidence rather than as an SCI-validated regulatory network, and each cross-context link requires direct testing in SCI models before it can be considered established.

## 8. Therapeutic Potential of Targeting the microRNA–Ferroptosis Regulatory Axis in Spinal Cord Injury

**Figure 3:**
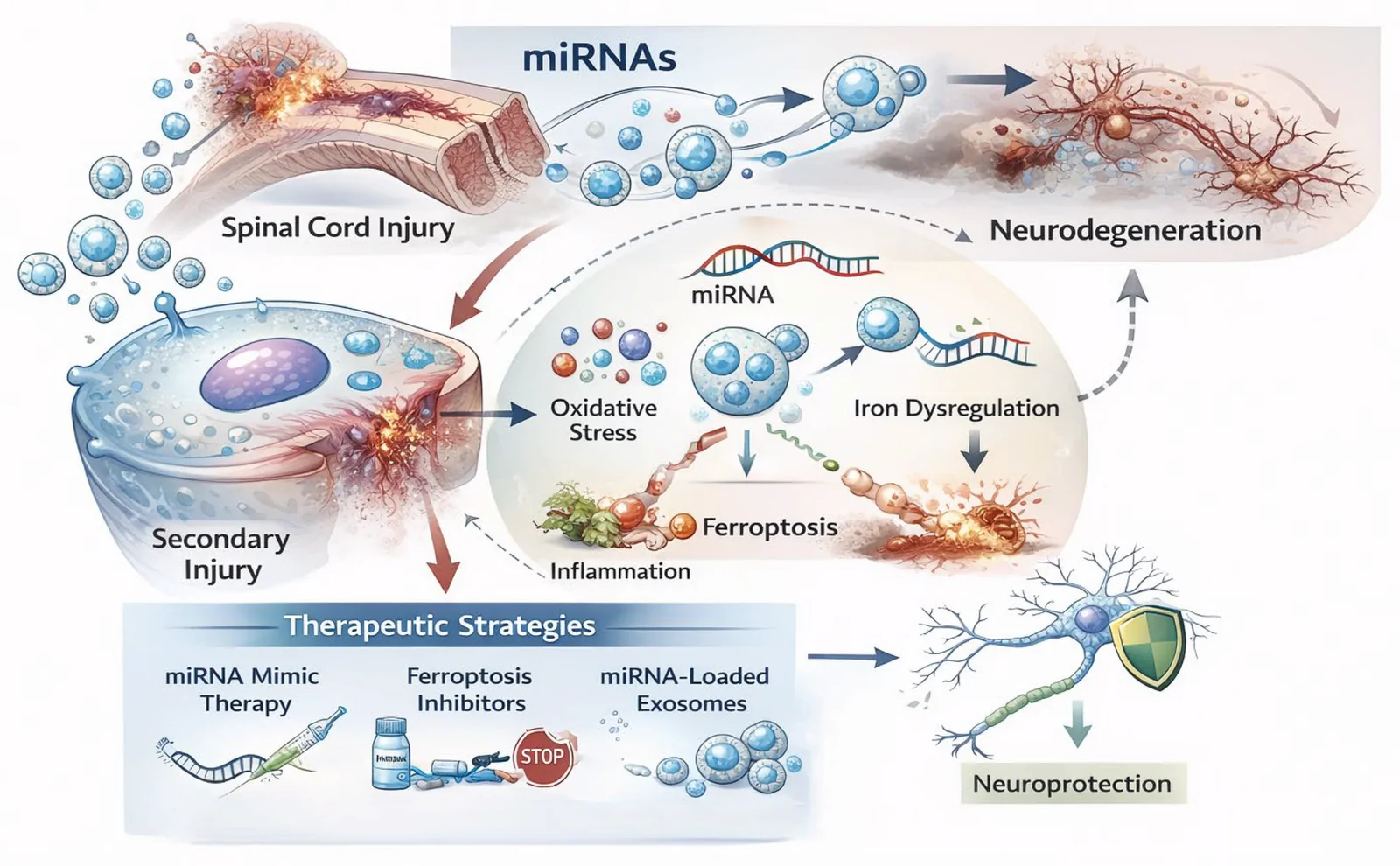
The microRNA–ferroptosis axis in spinal cord injury and its therapeutic targeting. Primary spinal cord injury (SCI) triggers secondary injury cascades, including oxidative stress, inflammation, and iron dysregulation. microRNAs (miRNAs) regulate key ferroptosis-related pathways by modulating genes involved in iron metabolism, lipid peroxidation, and antioxidant defenses. Excess iron accumulation and impaired redox homeostasis promote ferroptotic cell death, contributing to neuronal loss and neurodegeneration. Targeting the miRNA–ferroptosis axis represents a promising therapeutic strategy to attenuate secondary injury. Potential approaches include miRNA mimic therapy to restore protective miRNAs, ferroptosis inhibitors to suppress iron-dependent lipid peroxidation, and miRNA-loaded exosomes for targeted delivery. These interventions collectively aim to enhance neuroprotection and promote neural regeneration after SCI. Because the underlying studies report associated improvements in ferroptosis markers and functional outcomes rather than causal-isolation experiments that pinpoint ferroptosis as the operative mechanism, the specific contribution of ferroptosis suppression remains inferred rather than demonstrated. This schematic therefore depicts a proposed and biologically plausible pathway rather than one with causally established links at every node. Solid arrows indicate steps supported by direct experimental evidence in the cited studies; dashed arrows indicate links that are biologically plausible but not yet causally established.

Given the significant roles of both miRNAs and ferroptosis in the pathology of SCI, targeting the interplay between these two processes holds considerable therapeutic potential [5]. Several strategies are being explored to modulate miRNA expression to influence ferroptosis and promote neuroprotection and repair after SCI [99]. One approach involves the use of miRNA inhibitors, such as antagomirs or miRNA sponges, to block the action of miRNAs that promote ferroptosis, including miR-672-3p and miR-6315 [42]. Conversely, miRNA mimics could be employed to enhance the levels of miRNAs that are found to inhibit ferroptosis or promote neuroprotective effects (Figure 3; Table 5).

The first entry in Table 5, miR-19b-3p-enriched ADSC-derived exosomes, was tested in a mouse model of intracerebral hemorrhage rather than SCI. It is included here as proof-of-concept evidence that exosome-mediated miRNA delivery can inhibit ferroptosis and reduce neuronal damage in a CNS hemorrhagic injury setting, not as direct evidence of efficacy in SCI. Whether this delivery platform and target combination translates to SCI has not yet been tested.

Effective delivery of these miRNA modulators to the injured spinal cord is crucial for their therapeutic efficacy [99]. Various delivery methods are under investigation, including viral vectors, nanoparticles, and EVs, which offer the potential for targeted delivery [5]. For instance, the implantation of scaffolds enriched with miRNA-loaded EVs has shown promise [45], and neuronal EVs containing specific miRNAs have been found to promote SCI repair [3]. MSC-derived EVs have also demonstrated the ability to modulate ferroptosis and enhance neurological recovery in preclinical models [103]. Furthermore, researchers are developing engineered EVs that can respond to the specific microenvironment of the injury site, such as being responsive to ROS, for more targeted therapeutic action [104]. The development of safe and efficient delivery systems remains a critical aspect of translating miRNA-based therapies to the clinic.

A limitation applies to the therapeutic strategies summarized in Table 5 and depicted in Figure 3. Because EVs and miRNA modulators exert broad, pleiotropic effects on inflammation, apoptosis, oxidative stress, and regeneration (Table 3), the underlying studies report only convergent improvements in ferroptosis markers and functional outcomes. None have performed causal-isolation experiments such as epistatic or reconstitution designs to disentangle ferroptosis suppression from these other mechanisms, so the causal role of ferroptosis in the observed functional benefits remains unestablished. Targeting the miRNA–ferroptosis axis holds therapeutic promise, but causal dependency on ferroptosis in SCI remains unestablished. The convergent biomarker and functional effects reported across these modalities should therefore be interpreted as associative rather than mechanistically confirmed pending dependency studies. A supplementary table (Table S1) is provided, listing every microRNA discussed in the main text, including those not tabulated in Table 4 and Table 5.

In addition to modulating miRNA expression, directly targeting the ferroptosis pathway with specific inhibitors is another promising therapeutic strategy for SCI. Applying iron chelators, such as deferoxamine, and lipid peroxidation inhibitors, like ferrostatin-1 and SRS-16-86, has shown beneficial effects in preclinical SCI models [11]. Treatment with these ferroptosis inhibitors has demonstrated improved recovery and reduced tissue damage in animal studies [12]. Despite the promising preclinical findings, significant challenges remain in translating these therapeutic strategies to human clinical trials. Further research is necessary to fully elucidate the complex interplay between miRNAs and ferroptosis in the context of SCI. It is also crucial to investigate post-SCI miRNA expression profiles in human cell lines or non-human primates to establish a more robust foundation for clinical applications [3]. Addressing the limited understanding of the long-term effects of miRNA modulation and potential off-target effects is also essential [5]. Additionally, the potential problems that may be encountered during the clinical translation of ferroptosis inhibitors need careful consideration [12]. However, significant challenges remain in translating these promising preclinical findings to human clinical trials, including the need for further research into the complex interplay of miRNAs and ferroptosis in SCI, understanding long-term and off-target effects of miRNA modulation, and addressing potential issues with clinical translation of ferroptosis inhibitors.

## 9. Ferritinophagy in Spinal Cord Neurons: Mechanisms and Therapeutic Implications

**Figure 4:**
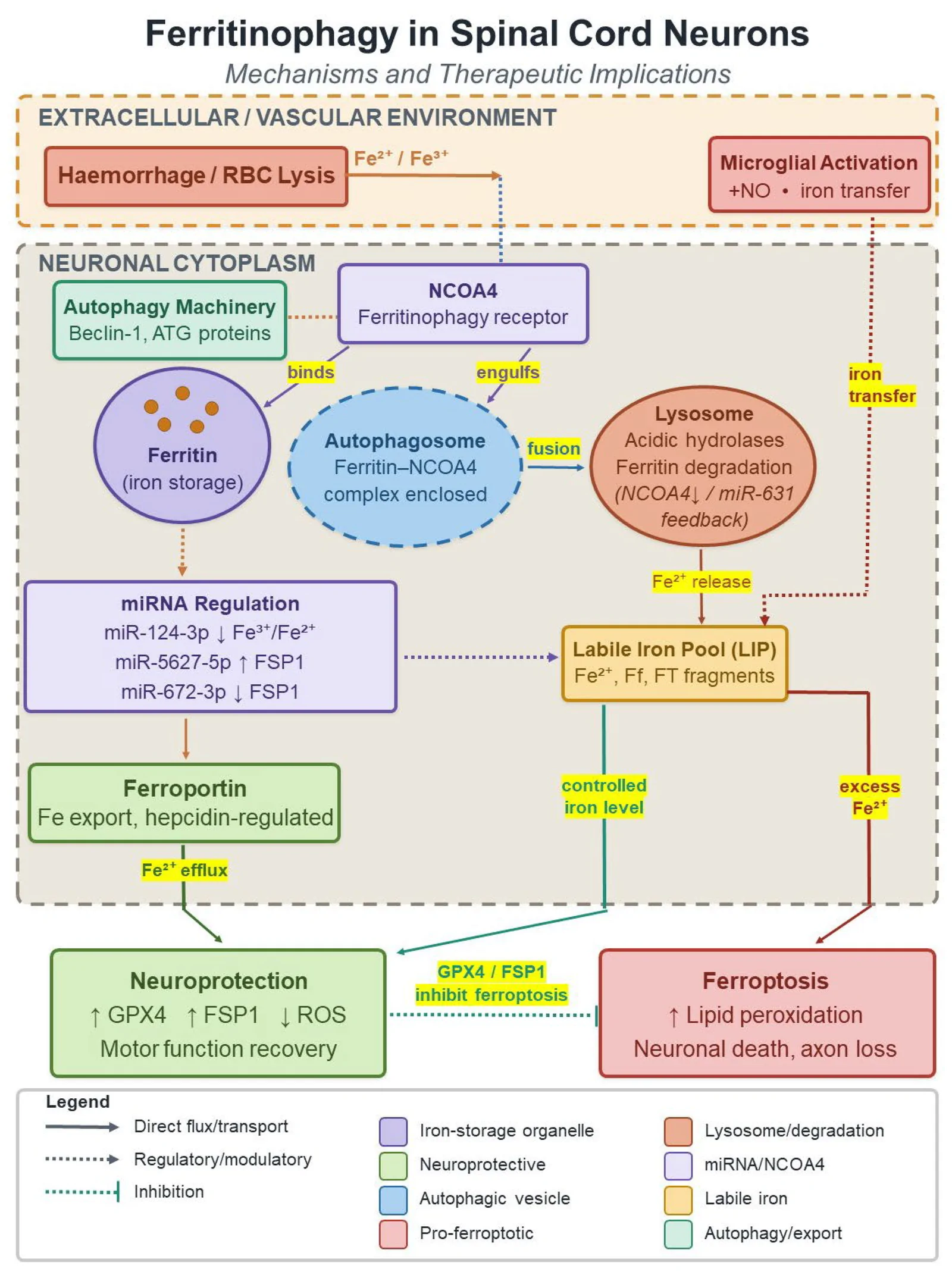
Mechanistic pathways of ferritinophagy and ferroptosis regulation in spinal cord neurons after spinal cord injury (SCI). Extracellular stressors following SCI, including hemorrhage-induced red blood cell (RBC) lysis and microglial activation (via nitric oxide release and iron transfer), drive cellular iron influx. Within the neuronal cytoplasm, the nuclear receptor coactivator 4 (NCOA4) binds iron-storing ferritin, directing it to the autophagy machinery (Beclin-1/ATGs) for autophagosome encapsulation. Subsequent fusion with the lysosome leads to ferritin degradation and the release of free ferrous iron (Fe^2+^) into the labile iron pool (LIP). MicroRNA (miRNA) networks dynamically modulate this system by regulating iron levels and downstream pathways. Under homeostatic conditions, controlled LIP levels and robust antioxidant systems—glutathione peroxidase 4 (GPX4) and ferroptosis suppressor protein 1 (FSP1)—inhibit lipid peroxidation, supporting neuroprotection and motor function recovery. Conversely, excessive or dysregulated ferritinophagy overwhelms cellular sequestration and ferroportin-mediated Fe^2+^ efflux. This accumulation of excess Fe^2+^ drives the Fenton reaction and lipid peroxidation, ultimately precipitating ferroptosis, axonal loss, and neuronal death. Upward (↑) and downward (↓) arrows denote up- or down-regulation.

Ferritinophagy is a selective autophagic process that regulates iron availability by targeting ferritin, the major intracellular iron-storage protein, for lysosomal degradation [105]. Following SCI-induced cellular stress, ferritin binds to the autophagy receptor nuclear receptor coactivator 4 (NCOA4), which mediates the recruitment of ferritin to the autophagic machinery and promotes the formation of ferritin-containing autophagosomes. These autophagosomes subsequently fuse with lysosomes, where ferritin is degraded and iron is released in a controlled manner to maintain iron homeostasis (Figure 4).

Ferritinophagy plays a critical role in regulating iron homeostasis and ferroptosis susceptibility following SCI. By reducing the labile iron pool and limiting iron-driven lipid peroxidation, ferritinophagy may function as a protective mechanism that mitigates ferroptosis and supports neuronal survival [106]. By facilitating the removal of excess iron, it can decrease substrate availability for the Fenton reaction, thereby reducing oxidative damage and lipid peroxidation [107]. Consequently, induction of ferritinophagy has been proposed as a potential therapeutic strategy to protect neurons and other cells from ferroptosis-induced damage after SCI.

However, the role of ferritinophagy is context-dependent. Excessive or dysregulated activation may disrupt iron homeostasis, leading to uncontrolled iron release into the cytoplasm and contributing to cellular damage if sequestration or export mechanisms are insufficient [108]. Despite this possibility, current evidence more commonly supports its protective role rather than a direct contribution to pathology in SCI. The interaction between ferritinophagy and ferroptosis is dynamic. SCI-induced cellular stress promotes iron accumulation and oxidative damage, thereby enhancing ferroptosis. In response, ferritinophagy may be activated as a compensatory mechanism to restore iron balance and limit ferroptotic injury [109]. This interplay highlights ferritinophagy as a context-dependent regulator of neuronal fate and a promising therapeutic target for reducing ferroptosis-mediated damage and promoting recovery after SCI.

Overall, ferroptosis is a key contributor to neuronal damage in SCI. Ferritinophagy appears to play a predominantly protective role by modulating iron levels and oxidative stress. Nonetheless, its precise function remains complex and incompletely understood, emphasizing the need for more specific tools and detailed temporal and cell type-specific studies to fully elucidate its therapeutic potential.

## 10. Challenges and Future Directions

Despite significant progress in understanding the role of ferroptosis and its regulation in spinal cord injury, several critical challenges remain that hinder effective clinical translation.

### 10.1. Limited Understanding of Context-Dependent Ferritinophagy

Ferritinophagy exhibits a dual role in SCI, functioning as both a protective and potentially detrimental process depending on cellular context and timing. While moderate activation may reduce iron overload and oxidative stress, excessive or dysregulated ferritinophagy could increase cytosolic iron levels and exacerbate ferroptosis. A major challenge lies in defining the precise conditions under which ferritinophagy shifts from neuroprotective to neurotoxic. Future studies should focus on temporal and cell type-specific regulation of ferritinophagy in neurons, astrocytes, and microglia. Much of the current evidence comes from single time-point sampling in cell lines or acute rodent models, and no validated method yet exists to track ferritinophagy flux longitudinally or non-invasively in an injured human spinal cord, which limits confidence in extrapolating these findings toward a clinical treatment window. From a safety standpoint, pharmacological ferritinophagy inhibitors risk disrupting systemic iron homeostasis if delivered outside the injury site, since ferritinophagy also supports normal iron recycling in erythropoiesis and hepatic storage. Cell type-specific conditional genetic models, such as neuron-, astrocyte-, or microglia-restricted NCOA4 manipulation, combined with longitudinal multi-omic profiling across the acute, subacute, and chronic phases of SCI, represent a key future direction for resolving this dual role.

### 10.2. Incomplete Characterization of miRNA Networks

Although numerous miRNAs have been implicated in regulating ferroptosis, the overall regulatory network remains incompletely understood. Most studies focus on individual miRNAs, while the combinatorial and synergistic effects of multiple miRNAs are largely unexplored. In addition, inconsistencies between studies highlight the need for standardized experimental models and validation across different SCI conditions. Systems-level approaches and multi-omics analyses are required to construct comprehensive miRNA–ferroptosis regulatory networks. Methodologically, most studies rely on single-miRNA gain- or loss-of-function experiments without accounting for compensatory changes elsewhere in the network, and many predicted miRNA-mRNA interactions rest on bioinformatic target prediction rather than direct experimental validation. Heterogeneity in animal models, injury severity, and sampling time points across studies further complicate cross-study comparison. Single-cell and spatial transcriptomic approaches, combined with network pharmacology modeling, are needed to map miRNA–ferroptosis regulatory circuits with cellular and spatial resolution rather than at the level of whole spinal cord homogenate. A related and more specific limitation is that this review itself draws several of the miRNA–ferroptosis mechanistic links summarized in Table 4 from non-SCI systems, including cancer cell lines, hemorrhage models, traumatic brain injury, and non-neural tissue, because SCI-specific validation is not yet available for these targets. Establishing which of these cross-context mechanisms operate in SCI, and to what degree, is itself a priority for future work, rather than an assumption the field can continue to make by analogy.

### 10.3. Challenges in miRNA Delivery and Stability

The therapeutic application of miRNAs is limited by challenges related to delivery, stability, and off-target effects. Efficient delivery across the BBB/BSCB, targeted cell specificity, and sustained expression remain major obstacles. While emerging delivery systems such as exosomes, nanoparticles, and viral vectors show promise, their safety, immunogenicity, and long-term effects require further investigation before clinical application. Naked miRNA mimics and antagomirs are rapidly degraded by serum ribonucleases and cleared renally within minutes, necessitating chemical modifications, such as 2′-O-methylation or phosphorothioate backbones, or encapsulation strategies to achieve therapeutically useful half-lives. Viral vectors such as AAV can provide sustained expression but raise safety concerns around insertional effects, pre-existing neutralizing immunity, and serotype-dependent tropism, while nanoparticle and exosome carriers face their own manufacturing, scale-up, and batch-to-batch consistency challenges that complicate regulatory approval. Resolving these translational barriers will require parallel progress in chemistry, formulation science, and good manufacturing practice-compliant production pipelines tailored specifically to CNS trauma indications.

### 10.4. Lack of Translational and Clinical Evidence

Despite these promising findings, research on miRNAs targeting ferroptosis in SCI remains largely preclinical, with most evidence derived from animal models. Clinical studies directly miRNA regulation of ferroptosis in human SCI are currently very limited and remain in the early stages of investigation. There is an urgent need for well-designed preclinical studies using clinically relevant models, followed by carefully controlled clinical trials to evaluate the safety and efficacy of ferroptosis-targeted therapies. A major translational barrier is the absence of standardized preclinical outcome measures that map cleanly onto clinical endpoints such as the ASIA Impairment Scale, making it difficult to judge which preclinical results are likely to generalize. Long-term safety data are essentially absent: the systemic consequences of chronically modulating a pathway as fundamental as ferroptosis, including possible effects on non-CNS tissues that rely on regulated iron-dependent cell death, remain unknown. Adopting rigor and reproducibility standards analogous to those developed for stroke research, alongside multicenter preclinical replication ahead of first-in-human, biomarker-guided adaptive trials, represents a plausible path toward clinical translation. This review’s evidence base contains a further gap: the functional and histological benefits reported for miRNA- and EV-based interventions are consistently observed alongside ferroptosis suppression, but they are never shown to depend on it. Because these interventions also modulate inflammation, apoptosis, and regeneration, ferroptosis inhibition cannot be isolated as the responsible mechanism from correlational data alone. To date, no published study has used a dependency or rescue design—such as co-administering a ferroptosis inducer or genetically restoring ferroptosis susceptibility—to establish this causal link. Until such experiments are performed, the miRNA–ferroptosis axis should be regarded as a promising but mechanistically unconfirmed therapeutic target in SCI.

### 10.5. Interaction with Other Cell-Death Pathways

Ferroptosis does not occur in isolation but interacts with other forms of regulated cell death, including apoptosis, necroptosis, and autophagy. The crosstalk between these pathways in SCI remains poorly understood. Future research should aim to elucidate how ferroptosis integrates into the broader cell death network and whether combinatorial therapeutic strategies targeting multiple pathways may yield improved outcomes. A key limitation is that the pharmacological tools used to define these death pathways (e.g., ferrostatin-1, necrostatin-1, pan-caspase inhibitors) show off-target and cross-pathway activity, and since inhibiting one pathway may simply redirect cells toward another, future work should combine multiplexed single-cell death pathway profiling with combinatorial inhibition strategies that account for this redundancy.

### 10.6. Need for Biomarkers and Therapeutic Windows

Reliable biomarkers for detecting ferroptosis and monitoring treatment response in SCI are currently lacking. Identifying specific molecular or imaging biomarkers would greatly enhance early diagnosis and therapeutic intervention. Additionally, defining the optimal therapeutic window for targeting ferroptosis and ferritinophagy is essential for maximizing neuroprotection. Current markers of ferroptotic activity, including tissue MDA, 4-HNE, and iron content, are generally measured invasively in animal spinal cord tissue and have no established, minimally invasive counterpart validated in human cerebrospinal fluid or blood, which is a substantial translational barrier. Imaging approaches such as quantitative susceptibility mapping, which can estimate tissue iron non-invasively, offer a promising avenue but have not yet been validated against histological ferroptosis markers in SCI specifically. Because the timescale of secondary injury differs between rodent models and humans, defining the therapeutic window will require biomarker validation directly in clinical cohorts rather than extrapolation from preclinical kinetics alone.

Across these six challenges, three recurring themes emerge. First, methodological heterogeneity in animal models, injury time points, and outcome measures limits the comparability and reproducibility of preclinical findings. Second, safety data are sparse for nearly every proposed intervention, whether a small-molecule ferroptosis inhibitor, a ferritinophagy modulator, or a miRNA-based therapeutic, and the systemic consequences of long-term pathway modulation remain largely unstudied. Third, translational barriers, including delivery across the BBB/BSCB, the absence of validated human biomarkers, and the mismatch between rodent and human injury timescales, recur across nearly all the mechanisms discussed in this review. Addressing these challenges will require coordinated progress in standardized preclinical models, delivery technologies, and biomarker development. This effort must also incorporate the rigor and reproducibility standards that have proven valuable in other areas of translational neuroscience before ferroptosis-targeted therapies for SCI can be responsibly advanced to clinical trials.

## 11. Conclusions and Future Perspectives

Accumulating evidence demonstrates that ferroptosis is a key contributor to secondary injury following SCI, driving neuronal and oligodendroglial loss through iron-dependent lipid peroxidation and oxidative stress. MiRNAs emerge as pivotal regulators within this process, orchestrating multiple ferroptosis-related pathways that influence cell survival, inflammation, and regenerative capacity. The growing recognition of the miRNA–ferroptosis axis provides new mechanistic insights into the molecular complexity of SCI pathophysiology. Despite significant progress, several challenges remain. Most current evidence is derived from preclinical models, and the temporal and cell type-specific roles of individual miRNAs in regulating ferroptosis after SCI require further clarification. In addition, effective and targeted delivery of miRNA-based therapeutics to injured spinal cord tissue remains a major translational barrier. Future studies integrating high-resolution transcriptomics, ferroptosis biomarkers, and functional outcomes will be essential to define clinically relevant targets. In summary, targeting the miRNA–ferroptosis regulatory network represents a promising avenue for mitigating secondary injury and enhancing neural regeneration after SCI. Targeting the miRNA–ferroptosis axis is promising, but a causal dependency on ferroptosis in SCI remains to be established, and the convergence of biomarker and functional effects reported across the studies discussed in this review should be read as associative rather than mechanistically confirmed. Continued mechanistic investigation and translational refinement may ultimately facilitate the development of novel therapeutic strategies aimed at improving functional recovery in patients with SCI.

## Figures and Tables

**Table 1:** MicroRNAs involved in ferroptosis regulation following spinal cord injury (SCI).

MicroRNAs	Expression Change After SCI	Ferroptosis-Related Target(s)	Effect on Ferroptosis	Functional Implication in SCI	Reference
miR-672-3p	Upregulated	FSP1	Promotes	Enhances neuronal ferroptosis and neurodegeneration	[36]
miR-6315	Upregulated	Smo, xCT, GPX4, GSH	Promotes	Exacerbates ferroptosis-mediated neuronal injury	[37]
let-7b-5p	Network-associated	p53 (indirect)	Promotes	Context-dependent role in neurodegeneration and repair	[3]
miR-15b-5p	Network-associated	Not determined	Not determined	Not determined	[38]

FSP1: Ferroptosis Suppressor Protein 1; Smo: Smoothened receptor; xCT: cystine/glutamate antiporter; GSH: Glutathione; GPX4: Glutathione Peroxidase 4; p53: protein 53.

**Table 2:** Temporal dynamics of ferroptosis activation following spinal cord injury (SCI). The table summarizes the temporal profile of ferroptosis after SCI, including key molecular events and biomarkers observed at different post-injury stages.

Time Post-Injury	Ferroptosis Activity	Key Features	Reference
0–6 h	Initiation	GSH depletion, early lipid ROS	[59]
6–24 h	Peak	GPX4 ↓, ACSL4 ↑, lipid peroxidation ↑	[60]
1–3 days	Sustained	Iron overload, mitochondrial damage	[60]
3–7 days	Declining	Recovery of redox balance begins	[60]
>7 days	Minimal	Scar formation, long-term injury processes	[58]

GSH: Glutathione; GPX4: Glutathione Peroxidase 4; ROS: Reactive oxygen species; Acyl-CoA synthetase long-chain family member 4. ↑: increased; ↓: decreased.

**Table 3:** Multifunctional effects of extracellular vesicles in ferroptosis regulation and spinal cord injury repair.

Category	Mechanism/Function	Biological Effect in SCI	References
Therapeutic Advantages	Safer and more controllable than MSCs; easier handling	Improved clinical applicability	[71]
Neuroprotection	Delivery of protective biomolecules	Enhances neuronal survival	[72]
Neuro-regeneration	Promotes axonal growth and repair	Facilitates nerve tissue regeneration	[73,74]
Scar Reduction	Modulates fibrotic responses	Attenuates glial scar formation	[75,76]
Oxidative Stress Regulation	Reduces ROS and oxidative damage	Limits secondary injury	[77]
Angiogenesis Promotion	Enhances vascular remodeling	Improves blood supply to injury site	[77]
BBB Restoration	Strengthens blood–brain barrier integrity	Prevents further damage and inflammation	[78]
Anti-apoptotic Effects	Inhibits apoptosis pathways	Reduces neuronal cell death	[79]
Anti-ferroptotic Effects	Regulates iron metabolism and lipid peroxidation	Suppresses ferroptosis	[80]
Drug Delivery Capability	Small size, high penetration, immune evasion	Efficient therapeutic cargo delivery	[81]
Mechanistic Action	Suppresses pathological responses; activates regenerative signaling	Supports neural and vascular repair	[78]
Combination Therapy	EVs + biomaterial scaffolds	Synergistic repair and nutrient support	[79,82]

**Table 4:** MicroRNAs regulating ferroptosis-related mechanisms in spinal cord injury and related neural injury models. The table presents microRNA–target interactions with established roles in ferroptosis regulation, drawn from spinal cord injury, related CNS injury, and non-neural or non-SCI systems (see Model system column); they are included because they act on ferroptosis regulatory nodes (GPX4, ACSL4, SLC7A11/xCT, ATF4, FSP1) that are broadly conserved across cell types, but the specific miRNA–target link has not yet been confirmed in SCI models for entries marked non-SCI.

miRNAs	Model System (Evidence Context)	Ferroptosis-Related Target(s)	Mechanism of Action	Cellular Outcome	References
miR-137	SH-SY5Y cells, hemorrhage exposure model (non-SCI)	SLC1A5	Regulates glutamate metabolism and redox balance	Modulates neuronal susceptibility to ferroptosis	[89]
miR-214	Hepatoma cells, erythroid cells (non-neural, non-SCI)	ATF4	Suppresses stress-induced ferroptosis signaling	Enhances neuronal survival	[90,91]
miR-212-5p	Traumatic brain injury, mouse (related CNS injury, non-SCI)	ACSL4	Reduces lipid peroxidation	Attenuates ferroptotic cell death	[92]
miR-200c	General/exercise physiology review (non-neural, non-SCI)	FSP1	Impairs antioxidant defense	Promotes ferroptosis and neuronal damage	[93]
miR-27a-3p	Solid tumor cells, drug resistance model (cancer, non-SCI)	SLC7A11 (xCT)	Inhibits cystine uptake and GSH synthesis	Increases ferroptosis sensitivity	[94]

SLC1A5: Solute carrier family 1 member 5; ATF4: Activating transcription factor 4; ACSL4: Acyl-CoA synthetase long-chain family member 4; FSP1: Ferroptosis suppressor protein 1; SLC7A11: Solute carrier family 7 member 11; xCT: Cystine/glutamate antiporter.

**Table 5:** Therapeutic strategies targeting the microRNA–ferroptosis axis in spinal cord injury. The table outlines experimental therapeutic approaches targeting microRNAs or ferroptosis-related pathways, including molecular targets, delivery strategies, and reported neuroprotective outcomes in preclinical studies; the experimental model column indicates whether each entry was tested directly in SCI or in a related non-SCI injury model (see note below).

Therapeutic Strategy	Molecular Target(s)	Delivery Method	Experimental Model	Effect on Ferroptosis	Functional Outcome	Reference
miR-19b-3p-enriched ADSC-derived exosomes	GPX4-related pathways	Exosome-based delivery	Intracerebral hemorrhage, mouse (non-SCI)	Inhibits	Reduces neuronal ferroptosis and tissue damage	[100]
Ferrostatin-1	Lipid peroxidation	Intraperitoneal injection	SCI	Inhibits	Attenuates neuronal death and improves functional recovery	[101]
Liproxstatin-1	Lipid peroxidation	Systemic administration	SCI	Inhibits	Reduces oxidative damage and neuronal loss	[102]
Iron chelators (e.g., deferoxamine)	Labile iron pool	Systemic administration	SCI	Inhibits	Decreases ferroptosis and secondary injury	[67]
miRNA modulation (mimics/inhibitors)	Ferroptosis-related genes	Viral or nanoparticle delivery	SCI	Context-dependent	Modulates neuronal survival and regeneration	[36]

SCI: Spinal cord injury.

## Data Availability

No new data were created or analyzed in this study. Data sharing is not applicable to this article.

## Supplementary Materials

The following supporting information can be downloaded at: https://www.mdpi.com/article/10.3390/ijms27177711/s1. References [110,111,112,113,114,115,116,117,118,119,120,121] are cited in the Supplementary Materials.

## Abbreviations

4-HNE	4-Hydroxynonenal
ACSL4	Acyl-CoA synthetase long-chain family member 4
ADSCs	Adipose-derived stem cells
Aifm2	Apoptosis-inducing factor 2
ATF4	Activating transcription factor 4
BBB	Blood–brain barrier
BSCB	Blood–spinal cord barrier
CNS	Central nervous system
CoQ10	Coenzyme Q10 (Ubiquinone)
Cpeb3	Cytoplasmic polyadenylation element-binding protein 3
CSF	Cerebrospinal fluid
DAMPs	Damage-associated molecular patterns
DFO	Deferoxamine (Iron chelator)
DNA	Deoxyribonucleic acid
EGCG	Epigallocatechin gallate
EndoMT	Endothelial-to-mesenchymal transition
Fenton reaction	Reaction in which ferrous iron (Fe^2+^) reacts with hydrogen peroxide (H_2_O_2_) to generate highly reactive hydroxyl radicals (•OH), which initiate lipid peroxidation and drive ferroptosis
FSP1	Ferroptosis suppressor protein 1
GPX4	Glutathione peroxidase 4
GSH	Glutathione
HMGB1	High-mobility group protein B1
IL-1β	Interleukin-1 beta
iPSC-NSCs	Induced pluripotent stem cell-derived neural stem cells
IRPs	Iron regulatory proteins
LIP	Labile iron pool
LncRNAs	Long non-coding RNAs
LOXs	Lipoxygenases
LRIG3	Leucine-rich repeats and immunoglobulin-like domains protein 3
MDA	Malondialdehyde
miRNAs	MicroRNAs
MK2	MAPK-activated protein kinase 2
mRNAs	Messenger RNAs
MSCs	Mesenchymal stem cells (or Mesenchymal stromal cells)
NCOA4	Nuclear receptor coactivator 4
NF-κB	Nuclear factor kappa B
NO	Nitric oxide
Nrf2/NRF2	Nuclear factor erythroid 2-related factor 2
OIP5-AS1	OIP5 antisense RNA 1 (LncRNA)
PUFAs	Polyunsaturated fatty acids
RISC	RNA-induced silencing complex
ROS	Reactive oxygen species
RSL-3	RAS-selective lethal compound 3
SCI	Spinal cord injury
SLC1A5	Solute carrier family 1 member 5
SLC7A11	Solute carrier family 7 member 11
Smo	Smoothened
TfR/TfR1	Transferrin receptor/Transferrin receptor 1
TLR4	Toll-like receptor 4
TNF-α	Tumor necrosis factor alpha
UBE2Z	Ubiquitin conjugating enzyme E2 Z
UTR	Untranslated region (e.g., 3′-UTR)
xCT	Cystine/glutamate antiporter sub-unit (related to System Xc-)
