# Supplementary material for: MicroRNA–Ferroptosis–Spinal Cord Injury: A Complex Interplay in Neurodegeneration and Repair
Source: Int J Mol Sci. 2026 Aug 28;27(17):7711. doi: 10.3390/ijms27177711 (PMC13565793; doi:10.3390/ijms27177711)
Supplement: Supplementary file 1 [file ijms-27-07711-s001.zip › ijms-4441385-supplementary.pdf]

Supplementary Table S1

Comprehensive listing of microRNAs discussed in the main text of this review "miRNA-Ferroptosis-Spinal Cord Injury: A Complex Interplay in Neurodegeneration and Repair", including entries not tabulated in Tables 4 and 5. For each miRNA, the table reports its molecular target(s) or pathway, the experimental context in which it was studied (cell type, animal model, or disease condition, including whether that context is SCI or a related/non-SCI system).

miRNA	Molecular Target(s) / Pathway	Context (cell type, model, or condition studied)	Reported Outcome	Ref.
**miR-9-5p**	TLR7/8 signaling (as endogenous ligand, non-SCI context; corrected citation)	Regulation of neuronal apoptosis after SCI	Contributes to apoptotic signaling	[1]
**miR-488**	HMGB1/TLR4/NF-κB pathway	Regulation of neuronal apoptosis after SCI	Inhibits neural inflammation and apoptosis	[2]
**miR-21**	Proliferation/apoptosis regulators in neural progenitor cells	Neural progenitor cell proliferation and apoptosis	Regulates both proliferation and apoptosis	[3]
**miR-124**	Apoptosis-related genes in neuronal cells	Neuronal apoptosis after SCI	Suppresses apoptosis; improves functional recovery	[4]
**miR-372**	Inflammatory signaling	SCI recovery	Implicated in inhibiting recovery	[5]
**miR-219**	Oligodendrocyte differentiation pathways	Remyelination after SCI	Promotes oligodendrocyte differentiation	[6]
**miR-543-5p**	Inflammation-related targets	Inflammatory/immune response after SCI	Regulates inflammation	[7]
**miR-940**	Inflammation-related targets; indirect ferroptosis links	Inflammatory/immune response after SCI; oxidative stress	Regulates inflammation	[8]
**miR-155**	M1 microglial polarization pathway	Microglial polarization after SCI	Pro-inflammatory; promotes M1 polarization	[9]
**miR-24-3p**	MAPK-activated protein kinase 2 (MK2)	Microglial activation after SCI	Downregulated after SCI; upregulation reduces microglial activation and TNF-α/IL-1β	[10]
**miR-146a**	Not specified	Spinal cord contusion	Upregulated following contusion	[11]
**miR-92a**	Cpeb3 (cytoplasmic polyadenylation element-binding protein 3)	Inflamed spinal cord neurons	Induced in inflamed neurons; targets Cpeb3	[12]
**miR-125a**	M1/M2 microglial polarization pathway	Microglial polarization after SCI	Regulates polarization; inhibits inflammatory response	[13]
**miR-216a-5p**	M1/M2 microglial polarization pathway	Microglial polarization after SCI	Regulates polarization; inhibits inflammatory response	[14]
**miR-129-5p**	HMGB1/TLR4/NF-κB pathway	Inflammation after SCI	Alleviates SCI by suppressing inflammation	[15]
**miR-99a**	Oxidative-stress-related targets	Oxidative stress after SCI (rat models)	Regulates oxidative stress	[16]
**miR-672-3p**	FSP1 (ferroptosis suppressor protein 1)	Ferroptosis after SCI	Upregulated; promotes ferroptosis and neurodegeneration by downregulating FSP1	[17]

miRNA	Molecular Target(s) / Pathway	Context (cell type, model, or condition studied)	Reported Outcome	Ref.
**miR-6315**	Smo, xCT, GPX4, GSH	Ferroptosis after SCI	Upregulated; promotes ferroptosis and exacerbates ferroptosis-mediated neuronal injury	[18]
**let-7b-5p**	p53 (indirect); ferroptosis-related mRNA-miRNA-lncRNA network	Ferroptosis network in SCI	Network-associated; context-dependent role in neurodegeneration and repair	[19]
**miR-15b-5p**	Not determined	Ferroptosis regulatory network in SCI	Network-associated; role not yet determined	[20]
**miR-26a**	Inflammation/apoptosis/oxidative-stress-related targets	Indirect ferroptosis modulation after SCI	May indirectly influence ferroptosis via established roles	[21]
**miR-5627-5p**	FSP1 (miR-5627-5p/FSP1 axis)	Neuronal ferroptosis after SCI, delivered via MSC-derived EVs	Inhibits ferroptosis; enhances resistance to oxidative death	[22]
**miR-9-3p**	Neuronal energy-metabolism-related targets	CSF extracellular vesicles after SCI	Candidate biomarker; adapts neuronal energy metabolism, reduces stress responses	[23]
**miR-133b**	RhoA pathway	Axonal regeneration after SCI (miRNA-loaded EV delivery, 200 μg dose)	Promotes axonal regeneration; optimal functional recovery in contusion models	[24]
**miR-219-5p**	UBE2Z/NRF2 pathway	Ferroptosis in neuronal cells after SCI (MSC-derived exosome delivery)	Reduces ferroptosis; improves motor function	[25]
**miR-125a-3p**	Neutrophil extracellular trap (NET) formation	Inflammation after SCI (exosome delivery)	Alleviates SCI by regulating NET formation	[26]
**miR-19b-3p**	GPX4-related pathways	Ferroptosis in intracerebral hemorrhage (ADSC-derived exosome delivery; non-SCI)	Reduces ferroptosis and neuronal damage	[27]
**miR-128-3p**	Nrf2 axis; SLC7A11 pathway	Ferroptosis regulation via lncRNA OIP5-AS1 (SCI and cancer contexts)	Suppresses ferroptosis; protects neural cells	[28, 29]
**miR-137**	SLC1A5 (glutamate metabolism)	SH-SY5Y cells, hemorrhage-exposure model (non-SCI)	Regulates glutamate metabolism and redox balance; modulates ferroptosis susceptibility	[30]
**miR-214**	ATF4 (stress-induced signaling)	Hepatoma and erythroid cells (non-neural, non-SCI)	Suppresses stress-induced ferroptosis; enhances survival	[31, 32]
**miR-212-5p**	ACSL4	Traumatic brain injury, mouse (related CNS injury, non-SCI)	Reduces lipid peroxidation; attenuates ferroptotic cell death	[33]
**miR-200c**	FSP1	General/exercise physiology review (non-neural, non-SCI)	Impairs antioxidant defense; promotes ferroptosis and neuronal damage	[34]
**miR-27a-3p**	SLC7A11 (xCT)	Solid tumor cells, drug-resistance model (cancer, non-SCI)	Inhibits cystine uptake and GSH synthesis; increases ferroptosis sensitivity	[35]

Reference

[1] X. He, J. Zhang, Y. Guo, X. Yang, Y. Huang, D. Hao, Exosomal miR-9-5p derived from BMSCs alleviates apoptosis, inflammation and endoplasmic reticulum stress in spinal cord injury by regulating the HDAC5/FGF2 axis, Molecular Immunology 145 (2022) 97-108.

[2] F. Niu, S. Pan, MicroRNA-488 inhibits neural inflammation and apoptosis in spinal cord injury through restraint on the HMGB1/TLR4/NF-κB signaling pathway, NeuroReport 32(12) (2021) 1017-1026.

[3] W.M. Zhang, Z.R. Zhang, X.T. Yang, Y.G. Zhang, Y.S. Gao, Overexpression of miR-21 promotes neural stem cell proliferation and neural differentiation via the Wnt/β-catenin signaling pathway in vitro, Mol Med Rep 17(1) (2018) 330-335.

[4] Z. Xu, K. Zhang, Q. Wang, Y. Zheng, MicroRNA-124 improves functional recovery and suppresses Bax-dependent apoptosis in rats following spinal cord injury, Mol Med Rep 19(4) (2019) 2551-2560.

[5] W. Zhou, T. Yuan, Y. Gao, P. Yin, W. Liu, C. Pan, Y. Liu, X. Yu, IL-1β-induces NF-κB and upregulates microRNA-372 to inhibit spinal cord injury recovery, Journal of Neurophysiology 117(6) (2017) 2282-2291.

[6] H.-B. Fan, L.-X. Chen, X.-B. Qu, C.-L. Ren, X.-X. Wu, F.-X. Dong, B.-L. Zhang, D.-S. Gao, R.-Q. Yao, Transplanted miR-219-overexpressing oligodendrocyte precursor cells promoted remyelination and improved functional recovery in a chronic demyelinated model, Scientific Reports 7(1) (2017) 41407.

[7] C.L. Zhao, H.A. Cui, X.R. Zhang, MiR-543-5p inhibits inflammation and promotes nerve regeneration through inactivation of the NF-κB in rats after spinal cord injury, Eur Rev Med Pharmacol Sci 23(3 Suppl) (2019) 39-46.

[8] B. Wang, P.F. Shen, Y.X. Qu, C. Zheng, J.D. Xu, Z.K. Xie, X.J. Cao, miR-940 promotes spinal cord injury recovery by inhibiting TLR4/NF-κB pathway-mediated inflammation, Eur Rev Med Pharmacol Sci 23(8) (2019) 3190-3197.

[9] X. Ge, P. Tang, Y. Rong, D. Jiang, X. Lu, C. Ji, J. Wang, C. Huang, A. Duan, Y. Liu, X. Chen, X. Chen, Z. Xu, F. Wang, Z. Wang, X. Li, W. Zhao, J. Fan, W. Liu, G. Yin, W. Cai, Exosomal miR-155 from M1-polarized macrophages promotes EndoMT and impairs mitochondrial function via activating NF-κB signaling pathway in vascular endothelial cells after traumatic spinal cord injury, Redox Biology 41 (2021) 101932.

[10] L. Zhao, J. Shen, K. Jia, F. Shi, Q. Hao, F. Gao, MicroRNA-24-3p Inhibits Microglia Inflammation by Regulating MK2 Following Spinal Cord Injury, Neurochemical Research 46(4) (2021) 843-852.

[11] Y. Tan, L. Yu, C. Zhang, K. Chen, J. Lu, L. Tan, miRNA-146a attenuates inflammation in an in vitro spinal cord injury model via inhibition of TLR4 signaling, Exp Ther Med 16(4) (2018) 3703-3709.

[12] I. Winkler, J.B. Engler, V. Vieira, S. Bauer, Y.H. Liu, G. Di Liberto, K.M. Grochowska, I. Wagner, J. Bier, L.C. Bal, N. Rothammer, N. Meurs, K. Egervari, B. Schattling, G. Salinas, M.R. Kreutz, Y.S. Huang, O. Pless, D. Merkler, M.A. Friese, MicroRNA-92a-CPEB3 axis protects neurons against inflammatory neurodegeneration, Sci Adv 9(47) (2023) eadi6855.

[13] Q. Chang, Y. Hao, Y. Wang, Y. Zhou, H. Zhuo, G. Zhao, Bone marrow mesenchymal stem cell-derived exosomal microRNA-125a promotes M2 macrophage polarization in spinal cord injury by downregulating IRF5, Brain Research Bulletin 170 (2021) 199-210.

[14] W. Liu, Y. Rong, J. Wang, Z. Zhou, X. Ge, C. Ji, D. Jiang, F. Gong, L. Li, J. Chen, S. Zhao, F. Kong, C. Gu, J. Fan, W. Cai, Exosome-shuttled miR-216a-5p from hypoxic preconditioned mesenchymal stem cells repair traumatic spinal cord injury by shifting microglial M1/M2 polarization, J Neuroinflammation 17(1) (2020) 47.

[15] G. Wan, Y. An, J. Tao, Y. Wang, Q. Zhou, R. Yang, Q. Liang, MicroRNA-129-5p alleviates spinal cord injury in mice via suppressing the apoptosis and inflammatory response through HMGB1/TLR4/NF-κB pathway, Biosci Rep 40(3) (2020).

[16] R. Wang, Y. Liu, L. Jing, MiRNA-99a alleviates inflammation and oxidative stress in lipopolysaccharide-stimulated PC-12 cells and rats post spinal cord injury, Bioengineered 13(2) (2022) 4248-4259.

[17] F. Wang, J. Li, Y. Zhao, D. Guo, D. Liu, S. Chang, H. Qiao, J. Li, Y. Yang, C. Zhang, R. Wang, F. Li, D. Wang, H. Li, X. He, miR-672-3p Promotes Functional Recovery in Rats with Contusive Spinal Cord Injury by Inhibiting Ferroptosis Suppressor Protein 1, Oxid Med Cell Longev 2022 (2022) 6041612.

[18] Z. Ma, Y. Fan, Y. Peng, L. Bian, J. Zhou, L. Wang, Y. Xia, S. Zheng, Y. Ji, Y. Han, C. Feng, Y. Ba, miR-6315 silencing protects against spinal cord injury through the Smo and anti-ferroptosis pathway, Bioscience reports 43(4) (2023) BSR20230030.

[19] J. Liu, G. Kong, C. Lu, J. Wang, W. Li, Z. Lv, J. Tong, Y. Liu, W. Xiong, H. Li, J. Fan, IPSC-NSCs-derived exosomal let-7b-5p improves motor function after spinal cord Injury by modulating microglial/macrophage pyroptosis, Journal of Nanobiotechnology 22(1) (2024) 403.
[20] H.-Y. Fu, Y. Li, H. Cui, J.-Z. Li, W.-X. Xu, X. Wang, R.-F. Fan, miR-15b-5p promotes HgCl2-induced chicken embryo kidney cells ferroptosis by targeting β-TrCP-mediated ATF4 ubiquitin degradation, Toxicology 503 (2024) 153742.
[21] X. Zhao, Y. Pan, S. Liang, Y. Lei, Y. Wang, L. Chen, Y. Zhao, M. Gan, L. Shen, X. Yang, L. Zhu, miR-26a-Targeting SLC7A11 Regulates Erastin-Induced Granulosa Cell Ferroptosis, Antioxidants (Basel) 14(11) (2025).
[22] C. Shao, Y. Chen, T. Yang, H. Zhao, D. Li, Mesenchymal Stem Cell Derived Exosomes Suppress Neuronal Cell Ferroptosis Via lncGm36569/miR-5627-5p/FSP1 Axis in Acute Spinal Cord Injury, Stem Cell Reviews and Reports 18(3) (2022) 1127-1142.
[23] T. Tanaka, S. Morimoto, K. Ito, K. Yasutake, C. Kato, M. Shinozaki, K. Suda, T. Maeda, Y. Yato, M. Nakamura, H. Okano, N. Nagoshi, Cerebrospinal fluid extracellular vesicle-derived miR-9-3p in spinal cord injury with neuroprotective implications and biomarker development, Communications Biology 8(1) (2025) 1498.
[24] M.-w. Liu, H. Li, G.-f. Xiong, B.-r. Zhang, Q.-j. Zhang, S.-j. Gao, Y.-l. Zhu, L.-m. Zhang, Mesenchymal stem cell exosomes therapy for the treatment of traumatic brain injury: mechanism, progress, challenges and prospects, Journal of Translational Medicine 23(1) (2025) 427.
[25] J. Dong, Z. Gong, H. Bi, J. Yang, B. Wang, K. Du, C. Zhang, L. Chen, BMSC-derived exosomal miR-219-5p alleviates ferroptosis in neuronal cells caused by spinal cord injury via the UBE2Z/NRF2 pathway, Neuroscience 556 (2024) 73-85.
[26] Y. Morishima, M. Kawabori, K. Yamazaki, S. Takamiya, S. Yamaguchi, Y. Nakahara, H. Senjo, D. Hashimoto, S. Masuda, Y. Fujioka, Y. Ohba, Y. Mizuno, Y. Kuge, M. Fujimura, Intravenous Administration of Mesenchymal Stem Cell-Derived Exosome Alleviates Spinal Cord Injury by Regulating Neutrophil Extracellular Trap Formation through Exosomal miR-125a-3p, International Journal of Molecular Sciences, 2024, p. 2406.
[27] X. Yi, X. Tang, Exosomes From miR-19b-3p-Modified ADSCs Inhibit Ferroptosis in Intracerebral Hemorrhage Mice, Frontiers in Cell and Developmental Biology Volume 9 - 2021 (2021).
[28] Z. Jiang, W. Zhang, J. Zhang, LncRNA OIP5-AS1 regulates ferroptosis and mitochondrial dysfunction-mediated apoptosis in spinal cord injury by targeting the miR-128-3p/Nrf2 axis, Heliyon 10(18) (2024) e37704.
[29] Y. Zhang, S. Guo, S. Wang, X. Li, D. Hou, H. Li, L. Wang, Y. Xu, B. Ma, H. Wang, X. Jiang, LncRNA OIP5-AS1 inhibits ferroptosis in prostate cancer with long-term cadmium exposure through miR-128-3p/SLC7A11 signaling, Ecotoxicology and Environmental Safety 220 (2021) 112376.
[30] Y. Li, J. Wang, S. Chen, P. Wu, S. Xu, C. Wang, H. Shi, J. Bihl, miR-137 boosts the neuroprotective effect of endothelial progenitor cell-derived exosomes in oxyhemoglobin-treated SH-SY5Y cells partially via COX2/PGE2 pathway, Stem Cell Research & Therapy 11(1) (2020) 330.
[31] T. Bai, R. Liang, R. Zhu, W. Wang, L. Zhou, Y. Sun, MicroRNA-214-3p enhances erastin-induced ferroptosis by targeting ATF4 in hepatoma cells, Journal of Cellular Physiology 235(7-8) (2020) 5637-5648.
[32] M. Gao, Y. Liu, Y. Chen, C. Yin, J.-J. Chen, S. Liu, miR-214 protects erythroid cells against oxidative stress by targeting ATF4 and EZH2, Free Radical Biology and Medicine 92 (2016) 39-49.
[33] X. Xiao, Y. Jiang, W. Liang, Y. Wang, S. Cao, H. Yan, L. Gao, L. Zhang, miR-212-5p attenuates ferroptotic neuronal death after traumatic brain injury by targeting Ptgs2, Molecular Brain 12(1) (2019) 78.
[34] A. Magenta, C. Cencioni, P. Fasanaro, G. Zaccagnini, S. Greco, G. Sarra-Ferraris, A. Antonini, F. Martelli, M.C. Capogrossi, miR-200c is upregulated by oxidative stress and induces endothelial cell apoptosis and senescence via ZEB1 inhibition, Cell Death Differ 18(10) (2011) 1628-39.
[35] X. Lu, N. Kang, X. Ling, M. Pan, W. Du, S. Gao, MiR-27a-3p Promotes Non-Small Cell Lung Cancer Through SLC7A11-Mediated-Ferroptosis, Front Oncol 11 (2021) 759346.
